# Cardiac Arrest Induces Ischemic Long-Term Potentiation of Hippocampal CA1 Neurons That Occludes Physiological Long-Term Potentiation

**DOI:** 10.1155/2018/9275239

**Published:** 2018-04-26

**Authors:** James E. Orfila, Nicole McKinnon, Myriam Moreno, Guiying Deng, Nicholas Chalmers, Robert M. Dietz, Paco S. Herson, Nidia Quillinan

**Affiliations:** ^1^Neuronal Injury Program, Department of Anesthesiology, University of Colorado, Anschutz Medical Campus, Aurora, CO 80045, USA; ^2^Department of Pediatrics, University of Colorado, Anschutz Medical Campus, Aurora, CO 80045, USA; ^3^Department of Pharmacology, University of Colorado, Anschutz Medical Campus, Aurora, CO 80045, USA

## Abstract

Ischemic long-term potentiation (iLTP) is a form of synaptic plasticity that occurs in acute brain slices following oxygen-glucose deprivation. *In vitro*, iLTP can occlude physiological LTP (pLTP) through saturation of plasticity mechanisms. We used our murine cardiac arrest and cardiopulmonary resuscitation (CA/CPR) model to produce global brain ischemia and assess whether iLTP is induced *in vivo*, contributing to the functionally relevant impairment of pLTP. Adult male mice were subjected to CA/CPR, and slice electrophysiology was performed in the hippocampal CA1 region 7 or 30 days later. We observed increased miniature excitatory postsynaptic current amplitudes, suggesting a potentiation of postsynaptic AMPA receptor function after CA/CPR. We also observed increased phosphorylated GluR1 in the postsynaptic density of hippocampi after CA/CPR. These data support the *in vivo* induction of ischemia-induced plasticity. Application of a low-frequency stimulus (LFS) to CA1 inputs reduced excitatory postsynaptic potentials in slices from mice subjected to CA/CPR, while having no effects in sham controls. These results are consistent with a reversal, or depotentiation, of iLTP. Further, depotentiation with LFS partially restored induction of pLTP with theta burst stimulation. These data provide evidence for iLTP following *in vivo* ischemia, which occludes pLTP and likely contributes to network disruptions that underlie memory impairments.

## 1. Introduction

Ischemic long-term potentiation (iLTP) is an increase in excitatory synaptic strength that occurs immediately following oxygen and glucose deprivation (OGD) in acute brain slices [1–6]. Elevations in extracellular glutamate during OGD cause prolonged activation of postsynaptic *α*-amino-3-hydroxy-5-methyl-4-isoxazolepropionic acid (AMPA) and N-methyl-D-aspartic acid (NMDA) receptors, resulting in an influx of sodium and calcium. Rises in intracellular calcium stimulate calcium/calmodulin-dependent protein kinase (CAMKII) signaling, which potentiates postsynaptic excitatory function via increased AMPA receptor phosphorylation and expression at the synapse. There is some indirect evidence to support that iLTP occurs following *in vivo* ischemia. Previously, we demonstrated increased activation of CAMKII within hours of global ischemia induced by cardiac arrest [7]. There is also evidence to support acute activation of CAMKII and increased NMDA receptor expression in the hippocampus within hours of *in vivo* focal ischemia [8]. However, it is unknown whether acute activation of CAMKII seen following *in vivo* ischemia causes synaptic potentiation in the hippocampus or whether ischemic LTP is maintained for days beyond the ischemic event.

Shared mechanisms between ischemic and physiologic LTP suggest that it is likely these plasticity processes would occlude one another, as has been described in studies where acute hippocampal brain slices were subjected to *in vitro* ischemia [3, 4]. Physiological hippocampal long-term potentiation (pLTP) is an experience- or frequency-dependent increase in synaptic strength and is a cellular substrate for learning and memory. Similar to iLTP, pLTP occurs through an NMDA and CAMKII-dependent increase in synaptic AMPA receptor function [9–12]. Memory deficits in cardiac arrest survivors are attributed to ischemic injury to the hippocampus that causes loss of pyramidal CA1 neurons [13, 14]. In addition to neuronal cell death, global ischemia causes persistent deficits in pLTP in surviving neurons of the CA1 [7, 15–19]. Therefore, pLTP deficits caused by brain ischemia likely contribute to memory deficits, and therapies that restore pLTP have the potential to improve cognitive function after CA/CPR. Acute neuroprotective interventions that reduce CA1 injury can also prevent pLTP deficits; however, there is no strategy that targets LTP deficits at delayed time points and that is independent of preventing neuronal cell death [7, 15, 19, 20]. The goal of this study was to determine whether *in vivo* global ischemia from cardiac arrest causes ischemic LTP that prevents physiological LTP.

## 2. Methods

### 2.1. Experimental Animals and Cardiac Arrest Model

The Institutional Animal Care and Use Committee (IACUC) at the University of Colorado approved all experimental protocols in accordance with the National Institutes of Health and guidelines for the care and use of animals in research. Analysis was performed with investigators blinded to experimental groups. Adult (8–12-week-old) male C57Bl6 (Charles River, Wilmington, MA) mice were subjected to CA/CPR as previously described during the ON light cycle [21–23]. A total of 54 animals were included in this study.

Briefly, anesthesia was induced with 3% isoflurane and maintained with 1.5–2% isoflurane in oxygen-enriched air using a nose cone. Temperature probes were inserted in the left ear and rectum to monitor tympanic (head) and body temperature simultaneously. A PE-10 catheter was inserted into the right internal jugular vein for drug administration. Needle electrodes were placed subcutaneously on the chest for continuous electrocardiogram (EKG) monitoring. Animals were endotracheally intubated and connected to a mouse ventilator (MiniVent Ventilator, Harvard Apparatus). Cardiac arrest was induced with injection of 50 *μ*l KCl (0.5 M) via the jugular catheter and confirmed by asystole on EKG. During cardiac arrest, the endotracheal tube was disconnected, anesthesia stopped, and body temperature was allowed to spontaneously decrease to a minimum of 35.5°C, and head temperature was maintained at 37.5°C. Resuscitation began eight minutes after induction of cardiac arrest by slow injection of 0.5–1.0 ml epinephrine solution (16 *μ*g epinephrine/ml 0.9% saline), chest compressions, and ventilation with 100% oxygen at a respiratory rate of 200 breaths/min. Chest compressions were stopped as soon as spontaneous circulation was restored. Resuscitation was abandoned if spontaneous circulation was not restored within 2.5 minutes. Mice were extubated after they recovered an adequate respiratory rate and effort. Sham controls underwent the same procedures as mice undergoing cardiac arrest including anesthesia, intubation, placement of the jugular catheter, EKG leads, and temperature management. Sham controls did not receive KCl or epinephrine injections or chest compressions. The animals were placed in a single-housed static recovery cage on a heated water blanket (35°C) for the first 24 hours of recovery and at ambient room temperature for long-term recovery (up to 30 days). Mice received soft food and subcutaneous saline for 3 days after surgery and had free access to water and regular chow.

### 2.2. Acute Slice Preparation

Following CA/CPR or sham surgery, mice were anesthetized with isoflurane (3.5%) and transcardially perfused with ice-cold artificial cerebral spinal fluid (ACSF) containing (in mmol/l) 126 NaCl, 2.5 KCl, 2.5 CaCl_2_, 1.2 MgCl_2_, 1.2 NaH_2_PO_4_, 21.4 NaHCO_3_, and 11 D-glucose, bubbled with 95% O_2_/5% CO_2_ to maintain pH of 7.4. Mice were decapitated and brains were rapidly removed. Horizontal hippocampal sections (300 *μ*M) were cut in ice-cold ACSF using a VT1200S Vibratome (Leica, Buffalo Grove, IL, USA) and then maintained at room temperature for at least 30 minutes prior to recording.

### 2.3. Miniature Excitatory Postsynaptic Currents (mEPSCs)

Whole-cell recordings were performed at room temperature (22°C) in a submersion chamber and were continuously perfused with ACSF containing picrotoxin (PTX, 100 *μ*M) and tetrodotoxin (TTX, 250 nM). Recordings were obtained using borosilicate glass pipettes that were fabricated using a Flaming/Brown heat puller (Sutter Instruments, Novato, CA, USA) to a resistance of 2–4 MΩ. Internal recording solution contained (in mmol/l) 120 K-gluconate, 9 KCl, 10 KOH, 4 NaCl, 10 HEPES, 0.05 EGTA, 1 MgCl_2_, 4 Na_2_ATP, and 0.4 Na_2_GTP. Series resistance was <20 MΩ and did not change more than 20% during the experiment. Whole-cell voltage-clamp recordings were performed at a holding potential of −70 mV. Gap-free continuous recordings were acquired in 3-minute sweeps. Miniature events were identified using Clampfit software with template event detection, and mEPSC amplitude and frequency were quantified for each cell. To generate cumulative probability histograms events from all sham or CA/CPR, mice were pooled.

### 2.4. Extracellular Field Recording

For extracellular recordings, slices were transferred to an interface recording chamber that was continuously perfused with ACSF (1.5 ml/min) and warmed to 32°C. Extracellular field excitatory postsynaptic potentials (fEPSPs) recorded in the stratum radiatum were evoked with a bipolar stimulus electrode positioned in the stratum molecularae/luminaris to evoke glutamate release from Schaffer collaterals (0.05 Hz). Input-output curves were generated by increasing stimulus intensity in 10 *μ*A increments and recording fEPSP slopes. Stimulus intensity was adjusted to produce a fEPSP with a slope that was 50% of the maximum. A stable baseline fEPSP was recorded for 20 minutes before theta burst stimulation (TBS; 10 trains of 4–100 Hz pulses) was applied to Schaffer collaterals. fEPSPs were recorded for 60 minutes following TBS, and percent change from baseline was calculated for the last 10 minutes of the recording. Low-frequency stimulation (LFS) was delivered for 10 minutes (900 pulses at 0.5 Hz), and percent change from baseline was analyzed 20 minutes after LFS. Data were compressed to 1-minute averages, and the extent of LTP or depotentiation was measured as percentage of the baseline fEPSP slope during the last 10 minutes of the recording.

### 2.5. Western Blot Analysis

Following CA/CPR or sham surgery, mice were deeply anesthetized with isoflurane (3.5%), heads were decapitated and brains were rapidly removed. Hippocampi were isolated and rapidly frozen with 2-methylbutane on dry ice. Individual hippocampi were homogenized in sucrose buffer containing protease and phosphatase inhibitors using a PTFE tissue grinder in a glass tube. Homogenates were centrifuged at 1000 ×g for 10 minutes to remove cellular debris and nuclei. Supernatant was removed and spun at 10,000 ×g for 15 minutes. This supernatant was collected and spun at 100,000 ×g for 60 minutes, yielding a supernatant that contains the cytosolic cellular fraction (S3). The pellet (P2) was resuspended in triton buffer and then centrifuged at 32,000 ×g for 20 minutes, yielding a pellet (P4) that contains the postsynaptic density (PSD) fraction. This pellet was resuspended in N-PER buffer (Thermo Fisher, Waltham, MA) containing protease and phosphatase inhibitors. The PSD protein concentration was quantified using a BCA kit, and samples were diluted in 4x denaturing sample buffer to a final concentration of 1 *μ*g/*μ*l. Protein (20 *μ*g) was loaded onto a polyacrylamide gel for protein electrophoresis and transferred to a PVDF membrane. Membranes were blocked in Tris-buffered saline with Tween (TBS-T) containing 5% BSA or milk. Primary antibody incubations were performed overnight at 4°C and detected using horseradish peroxidase-conjugated secondary antibodies. Bands were visualized using a maximum sensitivity-enhanced chemiluminescence substrate with the ChemiDoc Gel Imaging System (Bio-Rad, Hercules, CA). Multiple antibodies were probed on each membrane by stripping with Restore Plus stripping buffer after chemiluminescent detection. Integrated volume of bands was normalized to beta-actin integrated volume for that sample. Normalized protein expression is presented relative to sham controls.

### 2.6. Statistics

For electrophysiology experiments, *n* indicates the number of recordings with no more than two recordings for a given experiment from a single animal. Data are presented as mean ± SEM. Statistical comparisons were made between two groups using Student's *t*-test and multiple groups using one-way analysis of variance (ANOVA) followed by Dunnett's post hoc comparison of groups relative to control. Statistical comparisons were performed using GraphPad Prism 7.0. Differences with a *p* value of <0.05 were considered significant.

## 3. Results

### 3.1. Increased AMPA Receptor Function following In Vivo Ischemia

**Figure 1:**
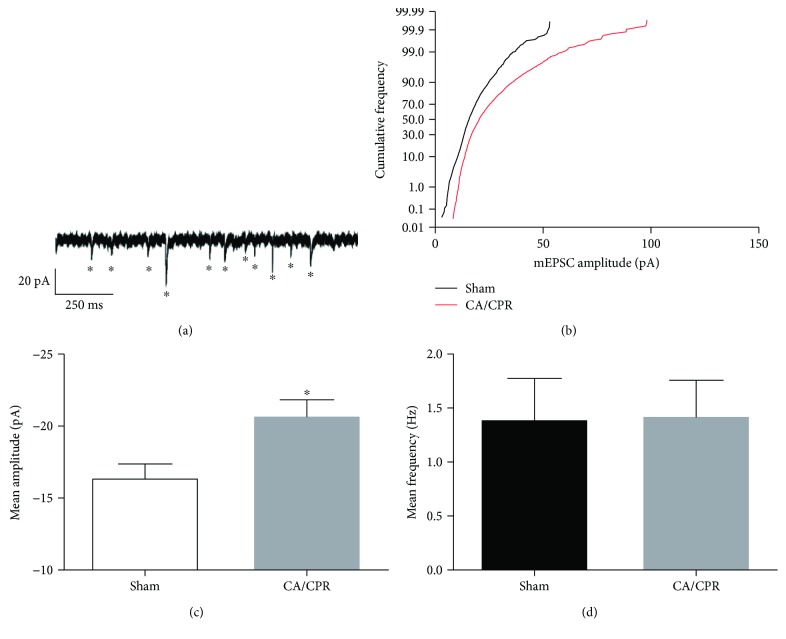
Lasting potentiation of miniature excitatory postsynaptic currents (mEPSCs) induced by cardiac arrest. (a) A representative trace from a sham control of whole-cell voltage clamp recording of mEPSC events recorded from CA1 neurons in acute brain slices. Events were detected with Clampfit software and are indicated with an asterisk. (b) CA/CPR produced a rightward shift in the cumulative frequency distribution of mEPSC amplitudes relative to shams. Events from sham (black, *n* = 2729 events) or CA/CPR (red, *n* = 3213 events) mice were pooled to generate histograms. (c) CA/CPR produced an increase in mean mEPSC amplitudes compared to sham. Mean mEPSC amplitude was calculated for each recording (sham: *n* = 12; CA/CPR: *n* = 15), and means for groups were compared using Student's unpaired *t*-test (∗ indicates *p* < 0.05). (d) CA/CPR did not alter synaptic density in CA1 neurons. No change in mean mEPSC frequency was observed between sham and CA/CPR mice. Mean mEPSC frequency was calculated for each recording (sham: *n* = 12; CA/CPR: *n* = 15), and means for groups were compared using Student's unpaired *t*-test.

The expression of LTP occurs through an increase in AMPA receptor function resulting from phosphorylation and increased synaptic expression. To directly measure postsynaptic AMPA receptor function, we performed whole-cell recording of miniature EPSCs (mEPSCs) in CA1 neurons 7 days after CA/CPR. Delayed neuronal cell death occurs at 2-3 days postinjury; therefore, by 7 days postinjury, cell death processes are complete and electrophysiology can be performed in surviving neurons that exhibit LTP deficits [7, 19, 22, 24]. Miniature excitatory events were isolated using tetrodotoxin (TTX, 250 nM) and picrotoxin (PTX, 100 *μ*M) (Figure 1(a)). Mean mEPSC amplitude, kinetics, and frequency were analyzed using Clampfit template event detection. Cumulative frequency distributions of mEPSC amplitudes were generated by pooling events from recordings in sham (*n* = 2,729 events) and CA/CPR (*n* = 3,213 events). The cumulative frequency curve was right-shifted in mice after CA/CPR compared to sham controls, with larger maximum amplitudes (110.7 pA versus 56.2 pA) (Figure 1(b)). The shift to larger events was also detected as an increase in the mean mEPSC amplitude from 16.43 ± 0.94 (*n* = 12) to 20.74 ± 1.1 (*n* = 15; *p* = 0.008) (Figure 1(c)). Rise and decay kinetics of mEPSCs were not different between shams and controls (Table 1). There were also no changes in the biophysical properties of neurons that would account for the larger amplitude mEPSCs observed after CA/CPR (Table 1). Event frequency was similar in sham (1.4 ± 0.4, *n* = 12) and cardiac arrest mice (1.4 ± 0.3, *n* = 15; *p* = 0.95) (Figure 1(d)), indicating no change in synapse number. These data suggest there is increased postsynaptic AMPA receptor function at CA1 synapses following CA/CPR.

**Figure 2:**
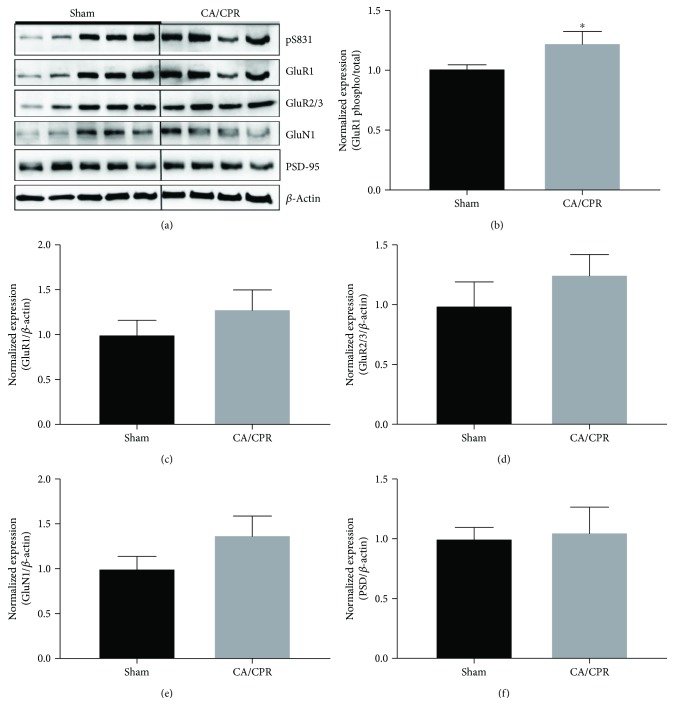
Increased AMPA receptor phosphorylation after CA/CPR. (a) Representative blots of protein expression from synaptic fractions of sham and CA/CPR hippocampus. Blots were cropped to show bands at molecular weight for indicated proteins. (b) Normalized phosphorylated S831: total GluR1 expression was calculated for each sample by dividing optical density of phosphoS831 by total GluR1 density within the same blot. (c) Normalized GluR1 expression was calculated for each sample by dividing optical density of total GluR1 by *β*-actin density within the same blot. (d) Normalized GluR2/3 expression was calculated for each sample by dividing optical density of total GluR2/3 by *β*-actin density within the same blot. (e) Normalized GluN1 expression was calculated for each sample by dividing optical density of total GluN1 by *β*-actin density within the same blot. (f) Normalized PSD-95 expression was calculated for each sample by dividing optical density of PSD-95 by *β*-actin density within the same blot. Values were normalized to sham controls. Shams (*n* = 7) and CA/CPR (*n* = 6) groups were compared using Student's *t*-test. ∗ indicates *p* < 0.05.

Synaptic potentiation results from NMDA receptor-dependent activation of CAMKII and the subsequent increase in AMPA receptor phosphorylation and expression at postsynaptic sites. Previously, we reported an acute increase in CAMKII activity (T286 phosphorylation) in the hippocampus 3-hour post-CA/CPR, suggesting an ischemia-induced increase in CAMKII activation [7]. To determine whether there are changes in glutamate receptor phosphorylation and expression at delayed time points after cardiac arrest, we isolated the hippocampus from shams and 7 days after CA/CPR, and protein fractions enriched for postsynaptic densities were subjected to Western blot analysis (Figure 2(a)). We observed an increase in levels of phosphorylated AMPA receptors (GluR1 pS831) from 1.01 ± 0.03 (*n* = 7) in shams to 1.23 ± 0.1 (*n* = 6) at 7 days postinjury (*p* = 0.047), consistent with an increase in receptor function (Figure 2(b)). GluR1 AMPA receptor expression (sham: 1 ± 0.16, *n* = 7; CA/CPR: 1.28 ± 0.22, *n* = 7) and GluR2/3 expression (sham: 1 ± 0.2, *n* = 5; CA/CPR: 1.25 ± 0.16, *n* = 4) were not different after cardiac arrest (*p* = 0.312 and *p* = 0.36, resp.) (Figures 2(c) and 2(d)). Ischemic LTP caused by *in vitro* ischemia can increase NMDA expression [8]. We observed a small increase in NMDA receptor (GluN1) from 1 ± 0.14 (*n* = 5) to 1.38 ± 0.21 (*n* = 4) expression that was not significant (*p* = 0.1675) (Figure 2(e)). Finally, we saw no change in PSD-95 levels after cardiac arrest (sham: 1 ± 0.1, *n* = 7; CA/CPR: 1.05 ± 0.21, *n* = 7; *p* = 0.823), suggesting no changes in the overall synapse density (Figure 2(f)). These data are consistent with our mEPSC data showing increased amplitude and no change in frequency of mEPSC events.

### 3.2. Depotentiation Restores the Ability to Induce Physiological LTP in Postischemic Neurons

**Figure 3:**
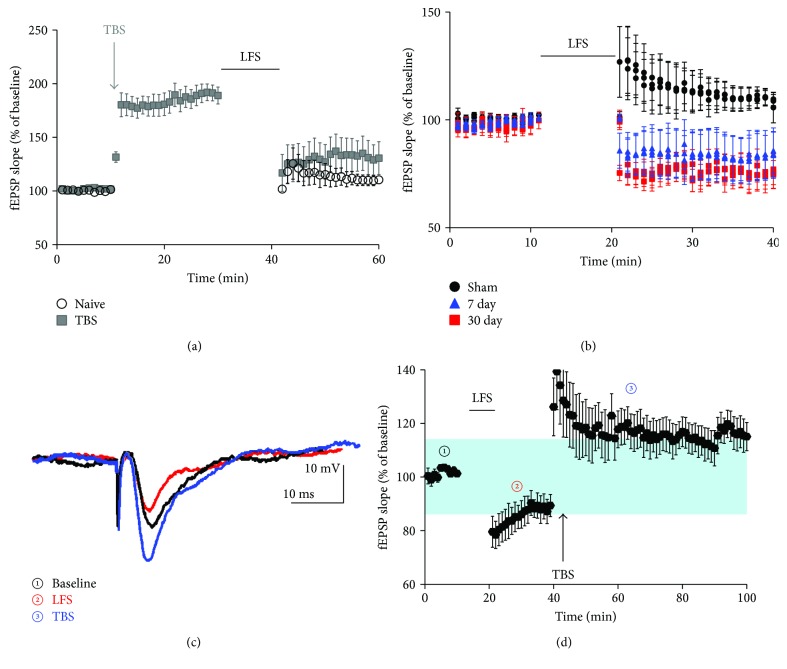
Depotentiation with low-frequency stimulation (LFS) reversed ischemic LTP and partially restored physiological LTP. (a) LFS depotentiates physiological LTP. 20 minutes after theta burst stimulation (TBS), LFS was delivered for 10 minutes (900 pulses at 0.5 Hz), resulting in a significant reduction in fEPSP slope (grey squares). LFS delivered to naive slices that did not receive TBS did not alter fEPSP slope (black circles). (b) LFS was delivered to slices from sham control (black circles) or 7 (blue triangles) or 30 days (red squares) after CA/CPR. LFS reduces fEPSC only in mice that were subjected to CA/CPR, indicating a reversal of iLTP. (c) Representative trace in recordings where we obtained a baseline (black trace) delivered LFS which reduced fEPSP amplitude (red trace) and subsequent TBS, which induced LTP (blue trace). (d) Summary of recordings in which we first delivered LFS then delivered TBS. Numbers on graph correlate with traces in panel (c). Magnitude of pLTP is shaded in blue.

Depotentiation, which is the reversal of LTP, is induced with low-frequency stimulation of synapses that were previously given high-frequency stimulation to induce LTP [25–28]. We hypothesized that ischemic LTP following CA/CPR would be reversed with a depotentiation stimulus. Previous studies use stimulation frequencies ranging between 0.5 and 2 Hz to depotentiate pLTP without inducing long-term depression (LTD). We found that 900 pulses, delivered at 0.5 Hz, reversed LTP that was induced by a previous theta burst stimulation (TBS) from 182.5 ± 7.7% to 132.7 ± 15.4% of baseline amplitude (*n* = 5). Importantly, this LFS protocol did not induce LTD in naive controls, having no effect on fEPSP slope from baseline following LFS (*n* = 6; *p* = 0.13) (Figure 3(a)), thus fitting the definition of a depotentiation protocol.

We next tested whether a depotentiation LFS protocol was capable of reducing synaptic strength in mice subjected to CA/CPR, thus providing further evidence of sustained iLTP. In sham controls, fEPSP slopes were 110 ± 4.2% (*n* = 6) of baseline after LFS, an increase that was not statistically significant (*p* = 0.06) (Figure 3(b)**)**. Acute slices prepared 7 days after CA/CPR showed a decrease in fEPSP slope to 83 ± 10.6% (*n* = 7) of baseline after LFS, a change that was not statistically different from baseline (*p* = 0.2) but was significantly different than the change observed in controls (*p* = 0.007). At 30 days after CA/CPR, the change in fEPSP slope to 75.73 ± 7.0% (*n* = 5) of baseline after LFS was significantly different from baseline (*p* = 0.015) and from the change observed in controls (*p* = 0.002). This provides additional evidence for CA1 synapses being in a potentiated state following *in vivo* ischemia.

The induction of ischemic LTP by CA/CPR may occlude physiological LTP. To test this, we delivered LFS to induce depotentiation and followed this with TBS in slices from mice at 7 days postinjury. After acquiring a stable 10-minute baseline, we delivered LFS, resulting in a decrease of fEPSP slope to 87.7 ± 0.4% (*n* = 5) of baseline (Figure 3(c), dotted line). After 20 minutes, we delivered TBS, which increased fEPSP slope to 115.1 ± 6.1% of original baseline, a potentiation of 28% (*p* = 0.015) (Figure 3(c), shaded blue). These data suggest that LTP mechanisms are saturated, and that reversal of ischemic LTP with LFS partially restores the capacity to induce physiological LTP.

## 4. Discussion

We have provided several pieces of evidence for the presence of sustained ischemic LTP subsequent to *in vivo* global ischemia caused by cardiac arrest: (1) increased postsynaptic glutamate receptor function, (2) increased postsynaptic glutamate receptor phosphorylation, and (3) the ability to depotentiate CA1 synapses after cardiac arrest. Further, we have shown that ischemic LTP occludes physiological LTP, providing a possible target for interventional strategies to improve memory function after cardiac arrest.

To our knowledge, this is the first study to demonstrate that *in vivo* ischemia causes synaptic alterations that are consistent with ischemic LTP. Until now, all electrophysiological evidence for this phenomenon comes from *in vitro* studies using oxygen and glucose deprivation in slices. Therefore, by showing that this phenomenon occurs in vivo, we suggest that this is a mechanism through which memory impairment occurs. Ischemic LTP is similar to physiological LTP in its NMDA receptor dependence, activation of intracellular signaling, and an increase in postsynaptic AMPA receptor function [3, 5]. The stimulus for inducing ischemic LTP is the massive increase in extracellular glutamate that occurs within minutes of the onset of ischemia [29–33]. Importantly, it is this massive increase in extracellular glutamate that stimulates excitotoxic cell death. By enhancing postsynaptic responses to extracellular glutamate, ischemic LTP likely amplifies excitotoxicity mechanisms [34, 35], but it is unclear from *in vitro* studies what contribution this phenomenon has to CA1 injury after CA/CPR. Similarly, it is difficult to disentangle ischemic LTP and excitotoxicity *in vivo*, as they have similar induction mechanisms. Previous work from our laboratory and others has shown that pharmacological or genetic interventions reduce NMDA receptor activation or CAMKII activation, not only reducing neuronal cell death but also preserving physiological LTP [7, 19]. It is possible that neuroprotective strategies prevent LTP impairments, in part, by blocking ischemic LTP.

Our strongest evidence for ischemic LTP comes from electrophysiological recordings that demonstrate increased miniature EPSC amplitude. The advantage of this method is that we can specifically assess postsynaptic receptor function in CA1 region of the hippocampus. In our recording conditions, increased mEPSC amplitudes likely represent increased AMPA rather than NMDA receptor function. Other groups have reported that iLTP observed at acute time points is a result of increased expression and function of NMDA receptors [1, 3, 8]. However, we have previously demonstrated no change in NMDA receptor function or expression at 7 days after CA/CPR, consistent with our results here [19, 20]. These differences may be due to the use of *in vivo* versus *in vitro* models, or that our studies were performed days, rather than hours after the ischemic insult. Analysis of glutamate receptor expression performed here was from the synaptic fraction of the entire hippocampus, not just the CA1 region. Therefore, increases in CA1 receptor expression may be underrepresented within this pool. Regardless, our Western blot data provided evidence for increased phosphorylation of the GluR1 AMPA receptor subunit, which is consistent with our electrophysiological data. There have been mixed results as to whether iLTP has a presynaptic mechanism [3, 6]. We failed to detect differences in paired-pulse ratio, suggesting a postsynaptic mechanism for iLTP induced by CA/CPR. Others have reported that impaired hippocampal LTP following global ischemia is associated with reduced spine densities [36–38]. However, we did not detect a reduction in mEPSC frequency and PSD-95 expression, which are indirect measures of the number of synapses. Therefore, our data is consistent with ischemia-induced changes in plasticity without changes in the number of functional synapses. However, further experiments are needed to rule out an ischemia effect on spine density that may contribute to impaired synaptic plasticity.

Physiological LTP and ischemic LTP have shared mechanisms and, therefore, have the ability to occlude one another. Indeed, tetanic stimulation delivered just prior to OGD prevents ischemic LTP and vice versa [4–6]. Remarkably, we saw that depotentiation prior to theta burst stimulation allowed for the induction of physiological LTP. Therefore, these results support in vitro findings that ischemic LTP saturates plasticity mechanisms to occlude physiological LTP. The stimulus frequency used to depotentiate had no effect on naive control slices, giving us confidence that we induced the depotentiation of synapses, rather than inducing long-term depression, which has different signaling mechanisms. While there was some physiological LTP following depotentiation, LFS did not restore completely back to naive control levels. Therefore, it is likely that there are additional mechanisms that contribute to the LTP impairments in the hippocampus after cerebral ischemia. Regardless, these data suggest that induction of depotentiation to restore physiological plasticity may be a relevant therapy for improving memory function after ischemic brain injury. Future studies should address whether *in vivo* low-frequency electrical stimulation of the hippocampus, with implanted electrodes or through transmagnetic stimulation, can produce depotentiation and reduce memory deficits *in vivo*.

*In vitro* studies have been limited in their ability to record ischemic LTP for only the first hours after ischemia. Here, we are able to show that ischemic LTP is maintained for weeks after injury onset. At 7 and 30 days of postinjury, cell death mechanisms have subsided and recordings are from the surviving hippocampal network. Our ability to depotentiate ischemic LTP and then induce physiological LTP at these delayed time points demonstrates that LTP impairments can be targeted to improve synaptic function, independent of acute neuroprotection. This is an important advance, as acute neuroprotective strategies have failed to improve cognitive outcomes in clinical trials. Cognitive impairments are present in patients that receive therapeutic hypothermia, the only strategy that has given positive results in cardiac arrest victims [39–41]. Therefore, strategies that can provide additional benefit to therapeutic hypothermia have promised to improve neurological function and quality of life for patients. Interestingly, rodent studies have shown that exposure of animals to novel environments can depotentiate previously acquired experience-dependent LTP, indicating the potential for novel rehabilitation strategies to reverse iLTP [28]. Future studies should determine whether such a behavioral paradigm could depotentiate ischemic LTP in the intact animal and improve future memory behavior.

In summary, we have demonstrated that *in vivo* global ischemia produces ischemic LTP which is the result of increased postsynaptic AMPA receptor function. Is iLTP beneficial or detrimental to hippocampal function? Our data demonstrating no change in input-output relations or synaptic density suggest that the hippocampal network is able to compensate for the loss of CA1 neurons after CA/CPR. Ischemic LTP may contribute to this normalization and therefore may have some benefit to the hippocampal network. However, the maintenance of iLTP for weeks after the ischemic insult is detrimental to physiological plasticity and likely worsens memory impairments. Thus, it appears that iLTP may serve as a beneficial compensatory mechanism following brain ischemia that if sustained during the chronic phase is detrimental to long-term recovery. Importantly, we show that this pathological form of plasticity is reversible and thus may be a therapeutic target for cognitive deficits after brain ischemia.

## Figures and Tables

**Table 1:** 

	Sham	7 days	30 days	*p* value
*R*_m_ (MW)	281.0 ± 30.85 (*n* = 10)	230.6 ± 38.65 (*n* = 16)		0.367
*C*_m_ (pF)	5.738 ± 1.360 (*n* = 10)	9.224 ± 1.845 (*n* = 16)		0.1893
PPR (pulse 1/pulse 2)	1.32 ± 0.1 (*n* = 5)	1.30 ± 0.08 (*n* = 8)	1.33 ± 0.07 (*n* = 4)	0.889
I/O (slope)	3.65 ± 0.59 (*n* = 6)	4.57 ± 0.33 (*n* = 6)	3.85 ± 0.78 (*n* = 6)	0.548
EPSC rise time (ms)	2.68 ± 0.27 (*n* = 12)	2.37 ± 0.17 (*n* = 15)		0.3271
EPSC decay time (ms)	11.60 ± 1.08 (*n* = 12)	12.55 ± 0.99 (*n* = 15)		0.5273
